# Potential for epidemic take-off from the primary outbreak farm via livestock movements

**DOI:** 10.1186/1746-6148-7-76

**Published:** 2011-11-24

**Authors:** Michael J Tildesley, Victoriya V Volkova, Mark EJ Woolhouse

**Affiliations:** 1 Centre for Immunity, Infection and Evolution, School of Biological Sciences, University of Edinburgh, Ashworth Laboratories, Kings Buildings, West Mains Road, Edinburgh, EH9 3JT, UK; 2 Epidemiology Group, Centre for Infectious Diseases, School of Biological Sciences, University of Edinburgh, Ashworth Laboratories, Kings Buildings, West Mains Road, Edinburgh, EH9 3JT, UK; 3 Centre for Complexity Science, Zeeman Building, University of Warwick, Coventry, CV4 7AL, UK (Current address; 4 Department of Population Medicine and Diagnostic Sciences, College of Veterinary Medicine, Cornell University, S2-064 Schurman Hall, Ithaca, New York 14853, USA (Current address

**Keywords:** livestock movement, seasonality, cyclicity, spread of infection, primary outbreak

## Abstract

**Background:**

We consider the potential for infection to spread in a farm population from the primary outbreak farm via livestock movements prior to disease detection. We analyse how this depends on the time of the year infection occurs, the species transmitting, the length of infectious period on the primary outbreak farm, location of the primary outbreak, and whether a livestock market becomes involved. We consider short infectious periods of 1 week, 2 weeks and 4 weeks, characteristic of acute contagious livestock diseases. The analysis is based on farms in Scotland from 1 January 2003 to 31 July 2007.

**Results:**

The proportion of primary outbreaks from which an acute contagious disease would spread via movement of livestock is generally low, but exhibits distinct annual cyclicity with peaks in May and August. The distance that livestock are moved varies similarly: at the time of the year when the potential for spread via movements is highest, the geographical spread via movements is largest. The seasonal patterns for cattle differ from those for sheep whilst there is no obvious seasonality for pigs. When spread via movements does occur, there is a high risk of infection reaching a livestock market; infection of markets can amplify disease spread. The proportion of primary outbreaks that would spread infection via livestock movements varies significantly between geographical regions.

**Conclusions:**

In this paper we introduce a set-up for analysis of movement data that allows for a generalized assessment of the risk associated with infection spreading from a primary outbreak farm via livestock movements, applying this to Scotland, we assess how this risk depends upon the time of the year, species transmitting, location of the farm and other factors.

## Background

The likelihood of epidemic take-off (spread from the primary outbreak farm to any other livestock holding) of an infectious disease introduced into a farm population is dependent upon a number of factors. Crucial to that are the pathways and chances for the infection to be transmitted from the primary outbreak to the rest of the population. Different types of contact between farms may result in transmission of infection [1-5]. However the movement of livestock has been observed to be the major route of spread of epidemics between farms during the period before the first farm is reported with disease (index case) - the "pre-detection" period. Examples include the two largest recent epidemics of contagious livestock diseases in the European Union - the outbreak of classical swine fever (CSF) in 1997-1998 (involving the Netherlands, Spain, Italy, Belgium and Germany) and the outbreak of foot and mouth disease (FMD) in 2001 (involving the UK, the Netherlands, France and the Republic of Ireland) [6-8]. Also, throughout the CSF outbreak the rate of between-herd transmission of infection via movements of pigs was much higher than that via any other route [5].

Lower and upper limits of the epidemic size in a farm network can be projected by calculating the numbers of farms in the giant strongly- and weakly-connected components, respectively, of the network. Using these measures, it has been shown that the possible final size of an epidemic in British livestock networks depends on the time of the year when the primary outbreak becomes infected for the cattle, sheep and mixed farm networks [9,10]. The epidemic size is also dependent upon the location of the primary outbreak [11,12]. In addition, a review of FMD outbreaks in all non-endemic areas of the world during 1992-2003 suggested that for this (characteristic acute contagious) disease the movement of infectious animals through a livestock market/auction at the start of an epidemic contributes the most to the epidemic's final size being exceptionally large (>2,000 infected premises) [13]. Therefore the early stages of an epidemic have a profound effect on the final number of farms infected and therefore its ultimate impact [11].

We introduce a framework to analyze livestock movements to evaluate an upper bound on the probability of spread of infection in a farm population from a primary outbreak farm via movements. Scottish farm networks are used as a model; the networks are evaluated from 1 January 2003 to 31 July 2007. A total of 27,400 farms appeared in the Scottish livestock networks from 1 January 2003 to 31 July 2007. Of these, 22,418 traded sheep, 15,634 traded cattle and 2,270 traded pigs, to/from other farms on at least one occasion. We consider both direct movements from the primary outbreak farm to other farms and movements that involve livestock markets. We analyse the dependency of each of these on the time of the year that infection occurs, the length of the infectious period (defined below) on the primary outbreak farm, and the species of livestock transmitting the disease.

In this paper, we focus on acute contagious livestock diseases. For these diseases, the length of time between the introduction of infection on the primary outbreak farm and the manifestation of clinical signs of disease in animals (disease detection) may vary. The primary outbreak farm remains "infectious" throughout the pre-detection period. The length of this period is dependent upon the virulence of the pathogen strain and the species infected. For example, the appearance of clinical signs of FMD is variable between species, with cattle and pigs typically showing signs far earlier than sheep. A 1 or 2 week farm-infectious period may be realistic when the primary outbreak species are cattle or pigs, whilst a 2 or 4 week period may be realistic for an outbreak starting in sheep. Moreover, for cattle, this would also depend on the strain of FMD virus. In the UK 2001 FMD outbreak, short pre-detection periods of < 10 days were generally observed. However in 1967 and 2007, pre-detection periods of up to 3 weeks were observed for a different strain of FMD virus. For CSF, an acute form of disease often manifests in 2-3 days, but, dependent on the strain, may take up to 2 weeks; a less virulent strain causing a sub-acute form of the disease may take even longer to detect. We therefore consider infectious periods on the primary outbreak farm of 1 week, 2 weeks and 4 weeks.

We then consider the number of farms in the first generation of an epidemic spreading through the livestock movement network. We also evaluate how the potential for spread of infection via movements varies between the regions of Scotland.

## Results

### Potential for spread of infection from the primary outbreak farm via livestock movements

**Figure 1:**
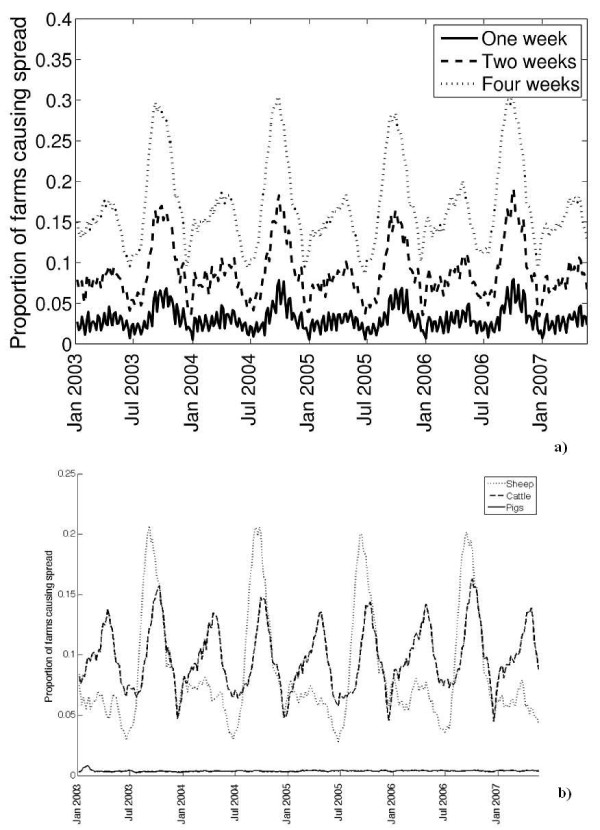
**Proportion of primary outbreak farms from which infection can spread to other farms via movement of (A) any livestock for infectious periods of 1, 2 and 4 weeks; (B) sheep, cattle and pigs for an infectious period of 4 weeks**.

In the event of an outbreak of an acute contagious livestock disease in Scotland, our results show that there would be no spread via livestock movements from a large proportion of primary outbreak farms. For a disease with a 1 week, 2 week and 4 week infectious period, 97%, 91% and 83% of the primary outbreaks, respectively, do not spread disease to other farms (Figure 1A). However the movement of livestock (as a whole) follows an annual cycle and peaks twice per year - in late April/early May and again in mid-August. Should the infectious period on the farm be 1 week, then the proportion of primary outbreak farms moving livestock to other farms while infectious varied from approximately 0.7% in late December to at most 8% in the second week of August, with a temporal average of 3%. If the primary outbreak was infectious for 2 weeks, this proportion ranged from 4% in late December to at most 19% in the second week of August, and on average was 9%. If the primary outbreak was infectious for 4 weeks, this proportion ranged from 9% in late December to 30% in mid-August, with a temporal average of 17%.

The seasonal patterns in the proportion of farms that can spread infection via livestock movements differed between species of livestock. The movement of sheep exhibited a single strong annual peak in mid-August (Figure 1B). At this time of the year, the infection would spread to at least 1 other farm via sheep movements from >20% of the primary outbreaks infectious for 4 weeks, whilst this value dropped to < 8% at all other times of the year. For cattle, the annual maximum of the proportion of farms with off-movements during a 4-week period was lower than for sheep. Also in contrast to sheep, this proportion for cattle peaked twice per year - in early May and in mid-August (Figure 1B). The peak in August tended to be slightly higher than that in May, with some year-to-year variation. During a 4-week infectious period commencing in mid-August, between 12% and 16% of primary outbreaks would have resulted in spread via cattle movement. The percentage of farms with off-movements of pigs was found to be the lowest of the three species, with spread occurring from < 0.5% of primary outbreaks at any given time of the year during a 4-week infectious period (Figure 1B). There was no obvious seasonality in pig movement.

### Potential for infection to reach a livestock market

**Figure 2:**
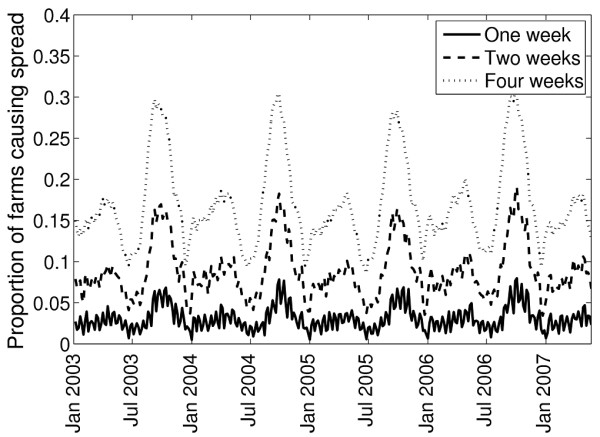
**Proportion of primary outbreak farms from which infection can spread to a livestock market via movement of any livestock for infectious periods of 1, 2 and 4 weeks**.

The proportion of primary outbreak farms from which infection can spread to a Scottish livestock market via animal movement peaked twice per year - in early May and in mid-August (Figure 2). This was comprised primarily of movements of sheep and cattle. The magnitude and annual cyclicity of the potential for spread to markets was close to the potential for any spread via movements (Figure 2 versus Figure 1A). No pig movements between Scottish farms via markets were recorded between January 2004 and July 2007.

### Numbers of farms in the first-generation of an epidemic

Of those primary outbreak farms that did move livestock off during the infectious period, the vast majority moved animals to only 1 farm. On the other hand, during a 1-week infectious period, on average 0.3% of primary outbreaks moved livestock to >4 farms (Table 1). This value rose to 1.1% for a 2-week infectious period and to 2.9% for a 4-week infectious period (Table 1). The maximum number of farms observed to become infected in the first generation varied significantly depending upon the primary outbreak farm and the time of the year infection occurred (for the case of a 4-week infectious period this maximum was around 200).

**Table 1:** Proportion of primary outbreak farms spreading infection to a given number of farms during the first generation of an epidemic (averaged over the period from 1 January 2003 to 31 July 2007) for a disease transmitted by any livestock depending on the length of the infectious period on the primary outbreak farm.

No. of farms in first generation	Proportion of primary outbreak farms causing spread for a given infectious period		
	1 week	2 weeks	4 weeks
1	0.0187	0.0478	0.0782

2	0.0054	0.0176	0.0335

3	0.0026	0.0085	0.0281

4	0.0017	0.0048	0.0124

> 4	0.0028	0.0105	0.0291

**Figure 3:**
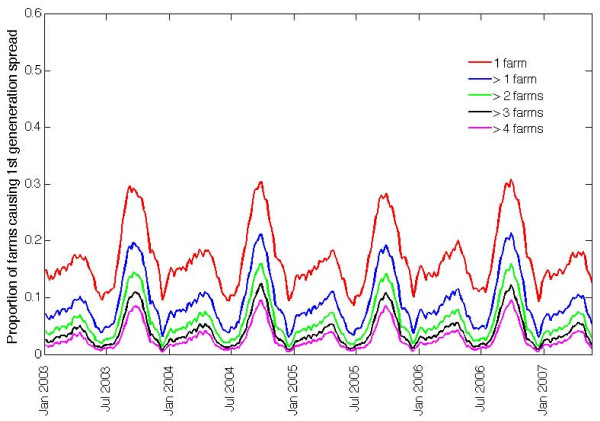
**Proportion of primary outbreak farms spreading infection to a given number of farms during the first generation of an epidemic for a disease transmitted any livestock and a 4-week infectious period on the primary outbreak farm**.

The seasonality in the numbers of farms in the epidemic's first generation was evaluated for a disease transmitted by all livestock with a farm-infectious period of 4 weeks. The seasonal patterns of the size of the first generation, in general, followed those of the potential for infection to spread via movements (Figure 3 versus Figure 1). The average likelihood of >4 farms becoming infected in the first generation was relatively low for most of the year, at 2.9%, but this increased to around 10% for epidemics commencing in mid-August.

### Regional differences

**Figure 4:**
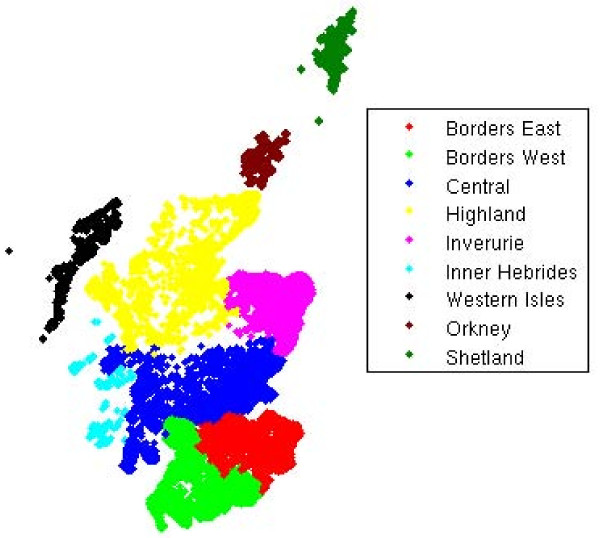
**Map of Scotland showing all livestock farms by region**.

**Figure 5:**
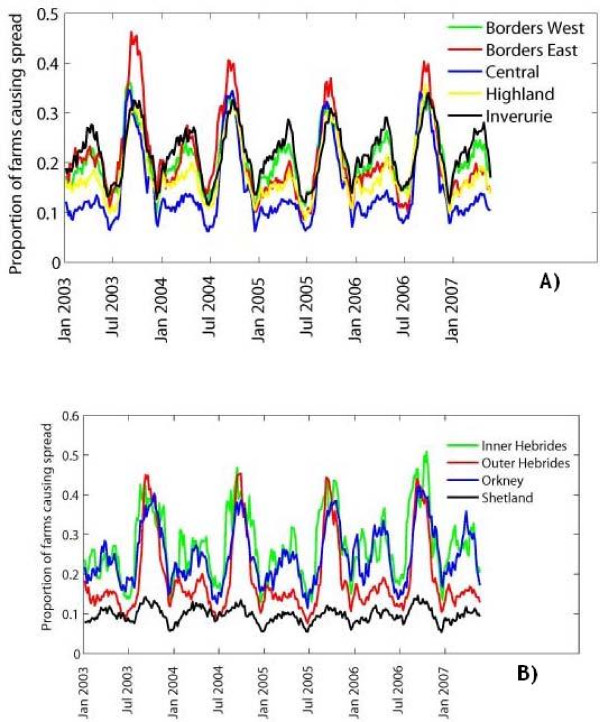
**Proportion of primary outbreak farms from which infection can spread to other farms via movement of any livestock for an infectious period of 4 weeks by region of Scotland: A) regions in mainland, and B) island archipelagos**.

The potential for infection spread via movements depended not only upon the time of the year infection occurred and the species transmitting, but also upon the location of the primary outbreak farm. We analysed how this potential varied between the nine regions of Scotland shown in Figure 4, for a disease transmitted by all livestock and the primary outbreak farm remaining infectious for 4 weeks. Overall, the seasonality of livestock movement was similar across the regions. The highest proportion of farms moving livestock off in any region was in mid-August, with a secondary peak in early May (Figure 5). However, there were differences in the magnitude of the proportion of farms from which infection can spread depending on whether the primary outbreak was located within the Scottish mainland or on a Scottish island (Figure 5A versus Figure 5B), as well as between individual parts of the mainland (Figure 5A) and between individual archipelagos (Figure 5B). The regions with the lowest risk were the archipelago of Shetland and the central part of the mainland, with a temporal average of 10% and 15% of primary outbreaks respectively resulting in spread. In the Highlands and on the Outer Hebrides archipelago the potential for spread averaged at 18%, whilst this value increased to around 21% in the East Borders, West Borders and Inverurie. The regions with the highest risk were the archipelagos of Orkney and Inner Hebrides, where on average 24% and 27% of primary outbreaks respectively resulted in spread. However, the total numbers of farms moving livestock on these two archipelagos represented a small percentage of the total for Scotland during the study period, with only around 1,000 farms on Orkney and 500 farms on the Inner Hebrides.

### Distance of movement

**Figure 6:**
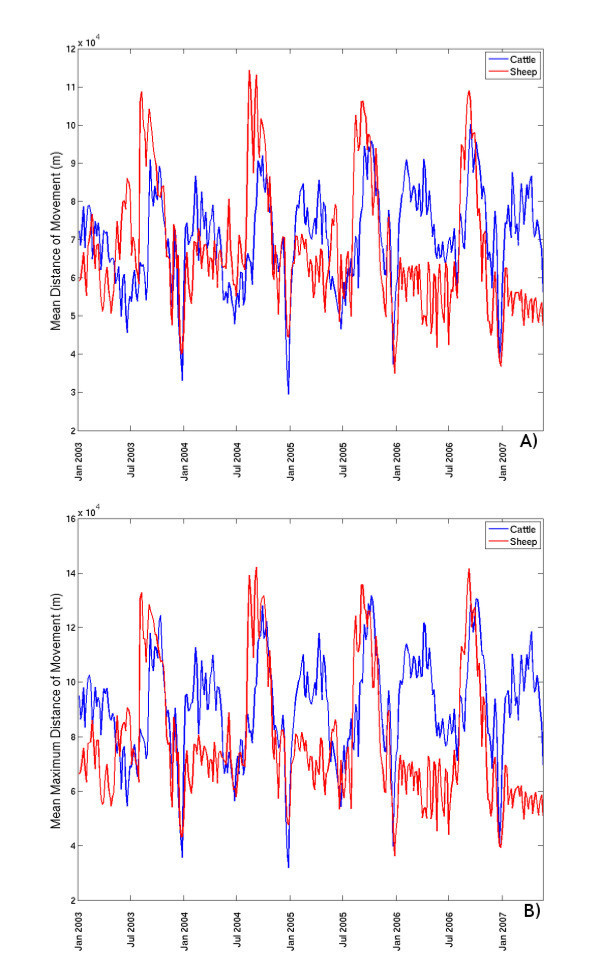
**A) Mean distance of movement and B) average (over all primary outbreak farms) of the maximum distance of movement during the first generation of an epidemic with a 2-week infectious period on the primary outbreak farm**.

Whilst the results presented above provide an indication of the potential for an epidemic to take off, an analysis of the distances that livestock travel during the epidemic's first generation provides further evidence of the risks associated with disease outbreaks. For the analysis of distances, we assumed that the infectious period on the primary outbreak farm was 2 weeks and investigated the distances travelled by cattle, sheep and pigs moved off the farm during this time. The results are summarised in Figure 6.

The mean distances travelled by cattle and sheep during the epidemic's first generation (with a 2-week infectious period) show cyclic annual behaviour similar to that of the potential for infection to spread via movements (Figure 6A; *cf*. Figure 1B). Dependent upon time of year, the mean distance of movement of cattle or sheep varies from 30 km to 120 km. Cattle travel furthest on average in late April/early May and in late August, with mean distances of 80-90 km and 90-100 km respectively (Figure 6A). Mean distances of sheep movement are ≤ 80 km for the first half of the year and peak at 100-120 km in mid-August (Figure 6A). The maximum distance of movement during the epidemic's first generation (with a 2-week infectious period), when averaged over all potential primary outbreak farms, closely mirrors the mean distance moved, with peaks of 100-120 km in April/May and 120-140 km in August for cattle, and 130-140 km in August for sheep (Figure 6B). However the maximum distance cattle and sheep may be moved during the epidemic's first generation is between 200 km and 650 km; there is no obvious seasonality in the maximum distance for cattle; for sheep it tends to peak between August and November. This indicates that, whilst average distances of movement are much lower, there is the potential for infection to spread across very significant distances prior to the detection of the outbreak.

No apparent annual cyclic behaviour is observed for distance of movement of pigs. When the infectious period on the primary outbreak farm is 2 weeks, the mean distance of movement during the first generation of an epidemic of a pig disease varies between 30 km and 80 km, whilst the maximum distance averaged over the primary outbreaks is slightly greater than this, at around 87 km. The maximum distance of pig movement varies between 100 km and 400 km. Whilst these maximum distances are significantly lower than in the cattle and sheep networks, there remains the potential for pig infection to be transmitted from the primary outbreak farm to a region several hundred kilometres away.

### Market-associated risk

**Figure 7:**
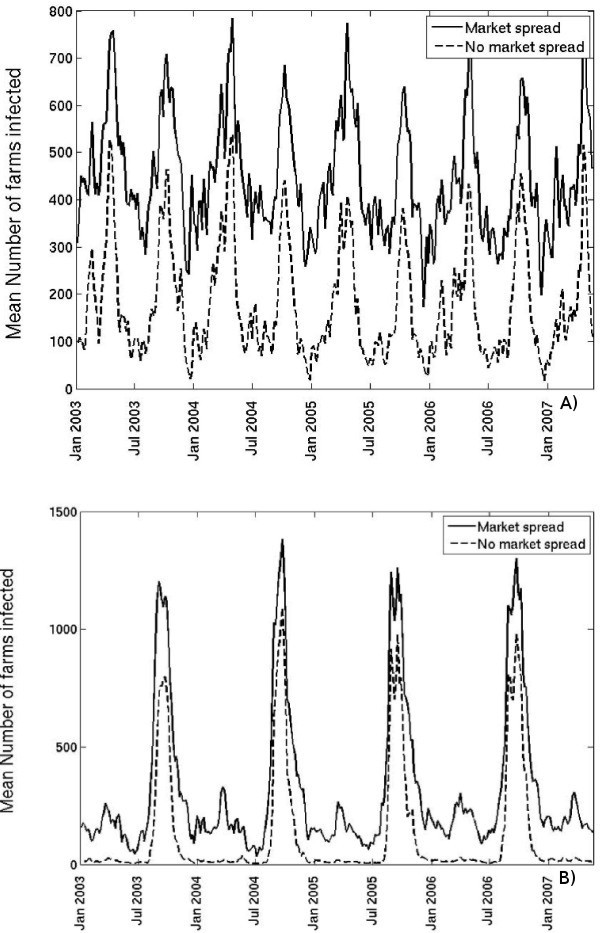
**Mean number of farms infected within 4 weeks of the primary outbreak farm becoming infectious, when a market amplifies transmission, versus a scenario in which only livestock from infected farms transmit disease for A) cattle and B) sheep, for a 2-week infectious period on the primary outbreak farm**.

All the results presented so far have made the assumption that when movements occur via markets, infection will be transmitted only to the farms buying livestock that originated from the primary outbreak farm. This ignored any amplification of transmission that may occur if the disease is transmitted to the other livestock on the market. This analysis also ignored subsequent spread from the infected farms (those that had received livestock from the infectious primary outbreak), *i.e*. the spread beyond the epidemic's first generation. In order to investigate the maximum potential for amplification of transmission by markets, we now assume that the market is infected upon receiving livestock from the primary outbreak farm, and all subsequent movements from that market result in infection being transmitted to all buying farms, for the length of the infectious period of the market (which is taken to be equal to the length of the infectious period on the primary outbreak farm). We analyse this scenario for both cattle and sheep, and for an infectious period of 2 weeks. The results for cattle are summarised in Figure 7A and for sheep in Figure 7B.

For a disease transmitted by cattle, if we assume that once a market is infected all movements from the market spread disease, the mean number of farms infected within 4 weeks of the primary outbreak farm becoming infectious is found to peak at 700-800 in late April/early May and at 600-700 in August (Figure 7A). However, if we assume that only movements from the infected farms transmit disease, the number of farms infected after 4 weeks is significantly lower, at around 400-550 farms in late April/early May and 400-500 farms in August (Figure 7A). For a disease transmitted by sheep, the peak in mid-August is also somewhat lower, at around 750-1100 farms when only movements from infected farms spread disease, compared with 1200-1400 farms when all movements from infected markets are infectious (Figure 7B). Perhaps more significantly, if we assume that only the livestock from infected farms spread disease, then for outbreaks commencing between January and July of each year, on average, fewer than 50 farms become infected, compared with up to 300 farms if all movements from infected markets are infectious (Figure 7B). These results imply that transmission of infection among animals at the market can amplify the spread of disease in the farm network. If the transmission at markets is likely to occur, the results in Figure 2, in which the proportion of primary outbreak farms spreading infection to a market is shown, provide a better indication of the likelihood of a large epidemic occurring. For a less contagious disease, for which the transmission at markets is unlikely, a better indication is the proportion of primary outbreaks spreading infection to any other farm, shown in Figure 1.

## Discussion

The primary aim of this paper is to assess the potential for epidemic to take off from a primary outbreak farm via livestock movements prior to disease detection. This potential is measured as the proportion of primary outbreak farms moving livestock to other farms whilst infectious. This analytical set-up is applied to Scottish livestock networks. The results demonstrate that the potential for epidemic take-off of a short-lived infection via livestock movements in this farm population is generally low throughout the year, averaging, for a disease transmitted by all livestock species, 0.03 when the animals on the primary outbreak remain infectious for 1 week, 0.09 for 2 weeks, and 0.17 for 4 weeks (Figure 1A).

Annual cyclicity of livestock movements is similar across regions of Scotland, but the maximum potential for infection spread at peak seasons and the average potential throughout the year differ (Figure 5). The latter was highest for primary outbreaks occurring on Orkney (housing 3.6% of the Scottish farms trading livestock during the study period), and the Inner Hebrides (housing 1.9% of the farms), while it was the lowest for primary outbreaks on Shetland, which alone housed 4.4% of the farms. Therefore, a high potential for spread of infection via movements can be seen for disease introductions into the regions with modest farm populations. Here we considered only the potential for spread via livestock movements; other modes of between-farm transmission of infection may depend on the numbers or densities of farms in the region [14,15].

The potential for any spread from the primary outbreak via movements, and the potential for infectious animals to be moved to a livestock market, appear to depend non-linearly on the length of infectious period on the farm (Figures 1 and 2). This may be because, under the standstill requirements, in many cases the movement of livestock off the farm is restricted for some days following an on-movement.

For a disease transmitted only by sheep, the proportion of primary outbreaks resulting in infection spread via movements peaks at a single point of the year - in mid-August (Figure 1B). For a cattle-only disease, this potential overall is lower, but also peaks in mid-August (with a secondary peak in early May; Figure 1B). Sheep movements in the other parts of Great Britain also peak in August [16]. However, when considered for the entirety of Great Britain between November 2001 and October 2003, cattle movements were observed to peak in April and October [17]. The seasonal adjustment of the surveillance effort to account for the peaks in cattle movements in Britain has been previously suggested [18]. A vigorous surveillance for contagious livestock diseases in Scotland at the peak of cattle and sheep movements in August may provide greatest benefit in terms of precluding significant pre-detection spread of infection between farms via livestock movements.

In August 2003, an outbreak of FMD occurred in Surrey, England. The farms in Scotland were not affected, but the restrictions imposed on the livestock movements due to the outbreak led to sizable consequences for the economy and for animal welfare [19,20]. According to our results, if the virus had been introduced to Scotland prior to detection of the outbreak, the likelihood of pre-detection spread via livestock movement would have been at its highest. This episode highlighted the importance of disease surveillance at this time of the year, but also the dilemma between the needs of disease control and the necessity for sufficient animal movement to take place to sustain the functioning of the industry.

The magnitude of the proportion of primary outbreak farms that can move infectious animals to a livestock market and its annual cyclicity are close to that for any spread from the primary outbreak via movements (Figure 2 versus Figure 1A). This is because 86% of sheep movements, 80% of beef and 42% of dairy cattle movements between Scottish farms during the study period occurred via markets, accounting for 79% of all the livestock movements. Therefore, even though the potential for infection to spread via movements from the primary outbreak in Scotland is generally low, if the spread does occur, it is likely via a market. A review of FMD outbreaks in non-endemic areas of the world between 1992 and 2003 showed that the movement of infectious animals through a market/auction at the beginning of an epidemic contributes to a large final epidemic size [13]. A decrease in the number of cattle movements between farms and markets over the entirety of Great Britain was reported for 2003 and 2004, compared to 2002 [21]. In Scotland on its own, the fraction of cattle movements via markets remained consistently high, from 78% to 82%, and the fraction of sheep movements varied from 83.5% to 88% each year from 2003 to 2007.

If we assume that once livestock is brought from the primary outbreak to the market, all subsequent movements from the market will transmit infection, in Figure 7 we see that there is the potential for disease to spread to several hundred farms within 4 weeks of the primary outbreak becoming infectious (if this remained infectious for 2 weeks). These results agree with a simulation study which showed that only small-size FMD outbreaks in Great Britain are possible without the amplification of transmission by an infected market [12]. Jointly, the results imply that in order to prevent large-scale disease outbreaks occurring in Britain, control emphasis should be placed upon the risk of spread from the primary outbreak to a market via movements of cattle or sheep.

In contrast, for a pig disease, the proportion of farms from which infection can spread via movements is very low throughout the year (< 0.005), with no obvious seasonal peaks, and no involvement of the markets. Therefore, a large-scale epidemic of a pig disease due to pre-detection spread via movements appears to be unlikely. The Scottish pig industry is a fattening industry; therefore the movements are normally confined within the breeder-fattening progeny pyramids, and happen only at certain points of the production cycle. This pyramidal structure of movements may account for the significant differences observed in the risks of epidemic take-off when compared with the cattle and sheep networks. However, our results need to be considered against the likely accuracy of data on pig movements. The movements within the pyramids may be under-reported owing to both source and destination holdings belonging to the same business. Our conclusions do agree however with those from a simulation study based on pig movements in Sweden, where from no to very limited spread was observed for an epidemic seeded to a fattening or a farrow-to-finish pig herd [22]. Also, in Belgian pig industries the farrow-to-finish herds were reported to have lower numbers of (farm-to-farm) movements compared to the other types of farms [4].

Another aspect of the risk of pre-detection spread of disease in a farm population via movements is the number of farms in the first generation of epidemic. This is defined here as the number of farms receiving livestock from the primary outbreak while it is infectious, and therefore measures the upper limit of the possible first generation, given no disease transmission at markets. In this analysis, the majority of primary outbreaks produce a first generation of 0 and a significant fraction produce a first generation of 1 farm. However, a small fraction of introductions can result in larger outbreaks. In rare cases up to 200 farms can be infected in the first generation (for an infectious period of 4 weeks). We speculate that such large first generations were observed for primary outbreak farms that were either dealing, or both farming and dealing livestock. Dealers have played a part in livestock movements in Scotland for decades [23]. A comprehensive identification of dealerships would allow efficient targeting of control to curtail the risk of pre-detection spread of epidemics via livestock movements. Also, seasonal sheep shows in Scotland can be held on farms (rather than on the registered show-grounds omitted from this analysis), although it is probably unlikely that the sheep would be returning to such large number of farms.

Whilst we consider scenarios of diseases transmitted by cattle, sheep or pigs separately, we do not analyse the risk of an epidemic occurring based upon the type of primary outbreak farm (*e.g*. breeder farm versus a feedlot for cattle). Number of contacts a pig holding makes was shown to depend on the holding type in Swedish [22] and Belgian [4] industries. It may be that in Scotland those livestock farms with greater number of contacts are breeding farms with high sanitary levels and biosecurity; this would decrease the risk of epidemic spread compared to our results. Coupling the movement records investigated in this analysis with the results of British agricultural censuses would allow for an analysis of the risk owing to farm type, and this is an aim for future research.

**Figure 8:**
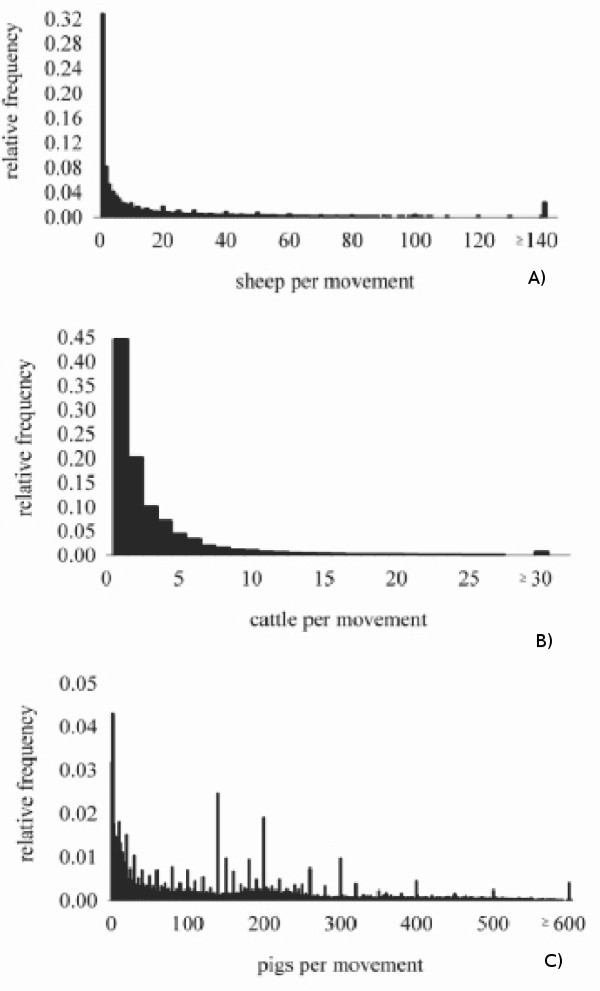
**Distribution of the number of animals per movement between Scottish farms of A) sheep, B) cattle and C) pigs from 1 January 2003 to 31 July 2007**.

In this analysis, the potential for spread of infection via livestock movement is evaluated as if any movement from the primary outbreak farm during its infectious period results in disease transmission to the recipient farm or market (we deterministically analyze the movement records). In reality, the dynamics of the infection on the primary outbreak will determine what proportion of its livestock becomes infectious and how rapidly. This, in combination with how many and which animals are being moved off, will determine what proportion of movements from the primary outbreak will contain infectious animals. Notably, in Scotland during the study period, the mode of the number of sheep (median = 4, mean = 19), cattle (median = 2, mean = 3) and goats (median = 2, mean = 4) per movement was 1 and the mode of the number of pigs per movement was 2 (median = 106, mean = 148). However the exact distribution of the number of animals per movement varied between the species; the distribution was somewhat diffuse for pigs, but highly over-dispersed and right-skewed for sheep and cattle (Figure 8). Therefore the results of our analysis provide upper bounds to the potential for infection to spread via movements in this farm population. For comparison, the field data suggest that during the pre-detection spread of FMD virus in Great Britain in 2001 just over half of the livestock movements from infected holdings to other farms resulted in transmission [12].

Our analysis is based on datasets from the systems that collated records of livestock movements (SAMS and CTS). A potential limitation is that, although the animal keepers (farmers, markets) were legally required to report the movements, it is possible that an unknown fraction of actual movements was not reported [1,24,25]. In addition, only around 90% of records of single movements of individual cattle from the CTS database were usable. Hence we may slightly under-quantify the numbers of livestock movements and the numbers of farms to which the animals were moved, and the upper bound of the risk of infection spread may be slightly higher than that suggested by our results.

The mean Euclidian distances cattle can travel during the first generation of an epidemic with a 2-week infectious period in Scotland at peak movement seasons are from 80 km to 100 km (Figure 6A). These are slightly larger compared to the average for Great Britain in 2001-2003 [17], and in 2004 [21], and sizably larger compared to median distance of cattle movements in the Netherlands in 2002-2003 [26], or mean or median distances of movements of cattle and sheep in New Zealand [27]. The mean distance of pig movements during a 2-week epidemic's first generation in Scotland can be between 30 km and 80 km. These are similar to the distances pigs are moved in Sweden [28], but are larger than the 19 km median distance of pig movements between farms in Belgium [4]. Hence there may be an opportunity for a larger geographical spread of a livestock infection prior to the detection of the outbreak in Scotland, compared to the livestock industries in continental Europe.

Further comparison of our observations to other parts of Europe highlights the similarities and differences in the rates and seasonal patterns of livestock movements between European territories. The double annual peak in cattle movement prior to and after the summer observed here for Scotland is also present in Sweden and in Italy [28,29]. However in Italy, in contrast to Scotland, cattle movements to markets increase from July to their annual maximum in October, and this post-summer peak is largely composed of cattle imports reared in other countries [29]. The median of 2 cattle per daily movement between two Scottish farms is close to that in Denmark, where it is 1 to 3 [26]; occasional movements of large numbers of cattle between farms also occur in both countries. The mean of 3 cattle per daily movement between two farms in Scotland is close to the 2.2 to 4.8 cattle per batch moving between farms in Italy [29]. The size of Scottish pig industry is small (total headage < 500,000 in each year 2003-2007 [20]) compared to some other European territories, *e.g*. Scandinavian, German or Dutch pig industries. However, we find that the uniform pattern of pig movements off Scottish farms throughout the year is similar to those reported in Denmark [30] and Sweden [28]. On the other hand, the median of 106 pigs per daily movement between two Scottish farms is fewer than that between two fattening holdings in Denmark, where it is 130-570 animals [30]. The patterns of livestock movements in individual regions of Europe are underlined by the conditions (climate, availability of pastures, etc) and traditions of livestock farming and trading. Nonetheless, the differences are likely to have implications for implementation of any common approaches to govern livestock movements to prevent or manage future epidemics. How these differences affect the potential for pre-detection spread of acute contagious livestock diseases via livestock movements can be quantified using analyses similar to those presented in this paper.

## Conclusions

We introduce a framework for analysis of livestock movement data to assess the potential for onward transmission of an acute contagious livestock disease from the primary outbreak farm via animal movements. It is applied to the Scottish farm networks, and the risk of epidemic take-off is assessed in this country-wide farm population in the absence of epidemic-associated restrictions on livestock movements. With an exotic disease in mind, this is the assessment of the potential for pre-detection spread of infection via movements. This complements information provided by methods for projecting the final epidemic size from the features of the farm network [9] or from the transmission potential of infection [11], which are only relevant once the epidemic has taken off.

## Methods

### Period of the study and livestock movement regulations

We aim to analyse livestock movements in the absence of epidemic-associated restrictions. This is analogous to considering the pre-detection period of an epidemic. The livestock movement restrictions associated with the FMD outbreak in the UK in 2001 were lifted in late 2002. The outbreak of FMD in Surrey in August 2007 led to restrictions of movements in Scotland until 31 December 2007. We therefore consider the period between 1 January 2003 and 31 July 2007. During this period, livestock movements on individual Scottish farms were subject to routine restrictions, known as "standstill". Standstill is a legally required period following a movement of livestock onto a farm during which no livestock may be moved from that farm to other farms (but livestock can be moved to slaughter). The legally required standstill period was 13 days following an on-movement of sheep, cattle or goats, and 20 days following an on-movement of pigs. However certain categories of movements were exempt from standstill, for example on holdings operating quarantine facilities.

### Livestock movement data and movement definition

The Scottish Animal Movement System (SAMS) is operated by Scottish Government. SAMS collates records of movements of batches of sheep, pigs and goats in Scotland. The records from 1 January 2003 to 31 July 2007 were extracted and processed using the Python programming language, and then in SAS^® ^9.1.3 software for Windows (SAS Institute Inc., Cary, NC, USA). The vast majority of the records were found to be logical and usable [24]. A batch of sheep, pigs or goats recorded to be moved from one Scottish holding to another on the same date was taken as a single movement. Multiple trucks could be used to transport a single batch of animals. The number of animals in each batch was available from the SAMS.

The Cattle Tracing System (CTS) of the British Cattle Movement Service is operated by the Department for the Environment, Food and Rural Affairs (DEFRA). The CTS collates records of single off-holding and on-holding movements of individual cattle in Great Britain. The records for Scotland from 1 January 2003 to 31 July 2007 were extracted. The records of movements of individual cattle for whom a logical movement history (*sensu lato *Mitchell et al. [17]) could be derived were separated for analysis. This resulted in the use of approximately 90% of the underlying individual-movement records. The data were summarized in a table where all cattle moved from one Scottish holding to another on the same date were considered as a single movement. The number of cattle per movement was counted. This table was assembled using the Python programming language; further data processing was done in SAS^® ^9.1.3 software for Windows.

The list of livestock markets, show-grounds, abattoirs and other industry units registered in Scotland was collated with help from the Livestock Traceability Policy Branch, Animal Health and Welfare Division, Scottish Government, and from the Animal Health agency in Scotland. This list was used to cross-check the types of holdings in the livestock movement records to insure that only movements between Scottish farms, directly or via Scottish livestock markets, were being analysed as such. Common-land premises were considered as farms. The records of the farm-(market)-farm movements were separated for analysis from both the SAMS and the table derived from the CTS. Other types of movements were excluded: from farms to abattoirs, between farms and registered show-grounds or insemination centres, movements from farms to/from non-farm holdings such as calf collection centres, between non-farm holdings, etc.

There may be a risk, theoretically, of infected livestock moving via a market to slaughter passing infection on to other livestock moving through that same market to farms. However, on Scottish markets the sheep and cattle designated for slaughter are normally sold on different days of the week from the sheep and cattle designated for breeding or further rearing (Roy Paterson, Scottish Government, and Andrew Wright, Institute of Auctioneers and Appraisers for Scotland, personal communication with Mike Lamont, Scottish Government). If these two groups of animals have to stay overnight on the same market, they are normally kept on different fields [31]. Effort is made to transfer the livestock to abattoirs as quickly as possible to prevent the animals losing condition [31]. Therefore, the risk of infection transmission from livestock sold via a market to slaughter to those sold through that market to farms is not considered in this analysis.

### Farm population

All Scottish farms (each defined by its unique holding-identifier) recorded (as per the movement data specified above) to trade sheep, cattle, pigs or goats to/from other Scottish farms at least once during the study period 1 January 2003 to 31 July 2007 were considered in this analysis. No further selection of the farms was done.

### Epidemic scenarios

**Figure 9:**
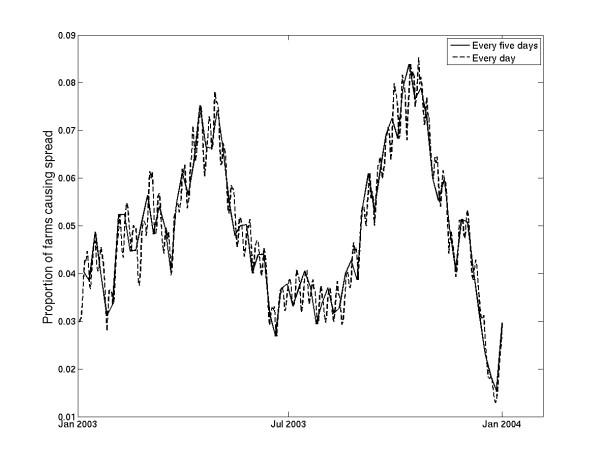
**Proportion of primary outbreak farms that transmit infection when infection is seeded every 5 days versus every day of 2003, for a disease transmitted by cattle with a 2-week infectious period on the primary outbreak farm**.

Epidemics are seeded every 5 days from 1 January 2003 to 31 July 2007. We analyze the movement network for each seeding date such that the primary outbreak varies across all farms in the population. That is, we deterministically analyse the movement data assuming that each farm becomes infectious (*i.e*. enters the infectious period as the primary outbreak) on each seeding date. Five-day interval between the seedings was chosen to reduce the computational time necessary to carry out this analysis. However, it is important to investigate whether any information is lost due to seeding every 5 days. We compared the effect of seeding every day as opposed to every 5 days on the observed patterns for the year 2003. Whilst some fine-scale daily variation is lost when seeding every 5 days, the overall annual cyclicity is the same as for every day seeding for all infectious periods considered; as an example, the comparison for an infection transmitted by cattle with a 2-week farm infectious period is illustrated in Figure 9.

The source of infection or its latent period is not considered in this analysis. The length of infectious period on the primary outbreak farm (the period for which its livestock remain infectious) is taken to be 1 week, 2 weeks or 4 weeks. A disease which can be transmitted by a single species (sheep, cattle or pigs), or by all livestock (sheep, cattle, pigs and goats) is considered. A scenario is defined by the length of infectious period on the primary outbreak farm and the species transmitting. The proportions of primary outbreak farms moving animals to any other farm or to a livestock market are calculated for every scenario for every seeding date.

The number of farms in the epidemic's first generation depending on the length of infectious period on the primary outbreak is calculated for an infection transmitted by all livestock species. The first generation is defined as those farms receiving livestock from the infectious primary outbreak farm. The seasonality in the size of the first generation is evaluated for the primary outbreak farm remaining infectious for 4 weeks.

The regional variation over Scotland in the proportion of primary outbreaks resulting in infection spread via livestock movements is evaluated for the scenario of a disease transmitted by all livestock species with a 4-week infectious period.

The mean and maximum Euclidian distances the livestock are moved during the epidemic's first generation are calculated for cattle, sheep and pigs, considering a disease where the primary outbreak farm is infectious for 2 weeks.

In order to investigate the potential risk of markets amplifying infection transmission during an epidemic in cattle or sheep, we estimate the number of farms infected within 4 weeks of the primary outbreak farm becoming infectious. Here we assume that the livestock brought from the primary outbreak to the market transmit infection to the rest of animals at the market, and all subsequent movements from the market result in transmission. Epidemics again are seeded every 5 days from 1 January 2003 to 31 July 2007 for a disease where the primary outbreak farm is infectious for 2 weeks, and the market remains infectious for 2 weeks after receiving animals from the primary outbreak. The results are compared against the assumption that infection is transmitted only by the livestock that are moved from the infected farms.

### Regions of Scotland

Each of the four Scottish island archipelagos has a distinct pattern of livestock movements throughout the year; within the Scottish mainland movements are also non-uniform [24,32]. One way to consider regionalization of livestock movement controls within Scotland is by the five divisional offices of Animal Health (acting as British governmental veterinary services). However, one of the offices oversees both the Orkney and Shetland archipelagos, and another incorporates the Western Isles. Moreover, the geographical coverage of each office does not exactly correspond to large-scale administrative borders. We therefore allocate the counties of the Scottish mainland into five regions, roughly corresponding to the mainland parts of the five offices, but consider each of the four Scottish island archipelagos on its own (Figure 4). We analyze livestock movements in each of these nine regions of Scotland.

## Authors' contributions

MJT, MEJW and VVV designed the study. MJT and VVV contributed equally to the study. VVV collated the movement data and prepared them for analysis. MJT implemented the simulations. MJT, MEJW and VVV interpreted the results. MJT and VVV drafted the manuscript. All authors read and approved the final manuscript.
